# Sequence optimized diagnostic assay for Ebola virus detection

**DOI:** 10.1038/s41598-023-29390-6

**Published:** 2023-11-01

**Authors:** Jeffrey W. Koehler, Christopher P. Stefan, Adrienne T. Hall, Korey L. Delp, Aileen E. O’Hearn, Cheryl L. Taylor-Howell, Nadia Wauquier, Randal J. Schoepp, Timothy D. Minogue

**Affiliations:** 1 Diagnostic Systems Division, United States Army Medical Research Institute of Infectious Diseases (USAMRIID), 1425 Porter Street, Fort Detrick, MD 20102 USA; 2 Metabiota, Kenema, Sierra Leone

**Keywords:** Biological techniques, Molecular biology, Infectious diseases, Microbiology, Infectious-disease diagnostics, Pathogens, Virology

## Abstract

Rapid pathogen identification is a critical first step in patient isolation, treatment, and controlling an outbreak. Real-time PCR is a highly sensitive and specific approach commonly used for infectious disease diagnostics. However, mismatches in the primer or probe sequence and the target organism can cause decreased sensitivity, assay failure, and false negative results. Limited genomic sequences for rare pathogens such as Ebola virus (EBOV) can negatively impact assay performance due to undiscovered genetic diversity. We previously developed and validated several EBOV assays prior to the 2013–2016 EBOV outbreak in West Africa, and sequencing EBOV Makona identified sequence variants that could impact assay performance. Here, we assessed the impact sequence mismatches have on EBOV assay performance, finding one or two primer or probe mismatches resulted in a range of impact from minimal to almost two log sensitivity reduction. Redesigning this assay improved detection of all EBOV variants tested. Comparing the performance of the new assay with the previous assays across a panel of human EBOV samples confirmed increased assay sensitivity as reflected in decreased Cq values with detection of three positive that tested negative with the original assay.

## Introduction

Ebola virus (EBOV), the causative agent of Ebola virus disease (EVD), is a zoonotic filovirus with high case fatality rate. EVD outbreaks are generally limited due to geographic isolation; however, the virus has high potential for person-to-person transmission through contact with infectious bodily fluids^1–3^. Large-scale outbreaks can occur when infected individuals are in densely populated regions with limited medical facilities such as in West Africa^4^ and the Democratic Republic of the Congo^2,3,5,6^. The primary strategy for containing EVD outbreaks historically focuses on rapidly identifying and isolating infected patients through effective diagnostics, maintaining rigorous infection control, and contact tracing (ex. health care workers, safe burials) measures to limit virus spread^7–9^. Recent incorporation of an EBOV vaccine into this strategy shows promise as an additional tool to contain an outbreak^10,11^. The rapid implementation of this comprehensive strategy initially appeared to slow the 2018 EVD outbreak in a densely populated region of the Democratic Republic of the Congo (DRC)^12,13^.

Real-time RT-PCR (RT-qPCR) is commonly used for infectious disease diagnostics due to high sensitivity, specificity, and ease of use. Care must be taken by the assay designers and the end-user to ensure the developed assay meets the intended use. Mismatches in the primer and/or probe compared to template can lead to significant impact on assay performance depending on the mismatch location^14^. Assay design for diagnostic purposes should incorporate all known pathogen sequences to ensure the assay will detect as much of the known diversity as possible while the end-user should ensure the assay is still valid based on current pathogens sequenced at the time of use.

One of the design challenges for rare infectious diseases such as EBOV is a limited known genetic diversity, which can negatively impact assay performance. Prior to 2013 there were 14 human EVD outbreaks caused by EBOV resulting in approximately 1500 infections with assays developed at the time using the limited genomic information available^15–18^. The largescale 2013–2016 EVD outbreak in West Africa resulted in thousands of EBOV infections and brought increased resources to address rare, high consequence pathogens. Rapid genomic sequencing used during the outbreak identified genomic diversity that could negatively impact previously developed assays^19^.

We previously developed and fielded a RT-qPCR assay, the Ebo-TM assay, targeting the EBOV glycoprotein, using the limited sequence information available at the time^15^. This assay was designed using EBOV Mayinga as the reference sequence. New sequence information, including the significant amount of new genomic data coming during and after the West Africa EBOV outbreak, identified mismatches between our primers/probe and different EBOV variants. Here, we assessed the impact the emerging sequence diversity had on assay performance. The assay tolerated a single mismatch well; however, two mismatches resulted in a 1–2 log decrease in assay sensitivity. Redesigning this assay to account for new sequence diversity improved assay sensitivity for all EBOV variants tested. A direct comparison using human EBOV clinical samples improved assay sensitivity resulting in several previously negative samples testing positive.

## Methods

### Human use and ethics

All methods were carried out in accordance with relevant guidelines and regulations, and all experimental protocols were approved by appropriate institutional and committee oversite and conducted in compliance with the United States Department of Defense, federal, and state statutes and regulations relating to the protection of human subjects, and adheres to the principles identified in the Belmont Report. USAMRIID Office of Human Use and Ethics reviewed this study and the de-identified samples and determined it to be Not Human Subject Research (HP-09-32). All samples were collected and de-identified in Sierra Leone at the Kenema Government Hospital, and the samples had indirect identifiers upon receipt. Samples were collected during the outbreak to perform emergency diagnostics and were not collected specifically for this study.

### Viruses and samples

Multiple EBOV variants were used in this study including Kikwit (UCC# R4317a-T; GenBank ID MK028855.1), Luebo (UCC# R4521T), Mayinga (UCC# R3831T), and Makona (UCC# R4491T, GenBank ID MH121161.1). All viruses are maintained by the Unified Culture Collection (UCC) at the US Army Medical Research Institute of Infectious Diseases (USAMRIID). Cell culture supernatant containing virus and human serum samples were inactivated using TRIzol LS (Thermo Fisher Scientific, Waltham, MA), and total nucleic acids were isolated using the EZ1 Virus Mini Kit V 2.0 (Qiagen, Valencia, CA) and the EZ1 Advanced XL (Qiagen) according the manufacturers’ recommendations. Samples were eluted in 90 µl elution buffer and stored at − 80 °C until use. A synthetic RNA (BioSyn, Inc., Lewisville, TX) encompassing the assay target region for EBOV Beni (GenBank# MK163673), which had a mismatch in the forward primer, was used for inclusivity testing.

### EBOV sequence analysis

**Figure 1:**
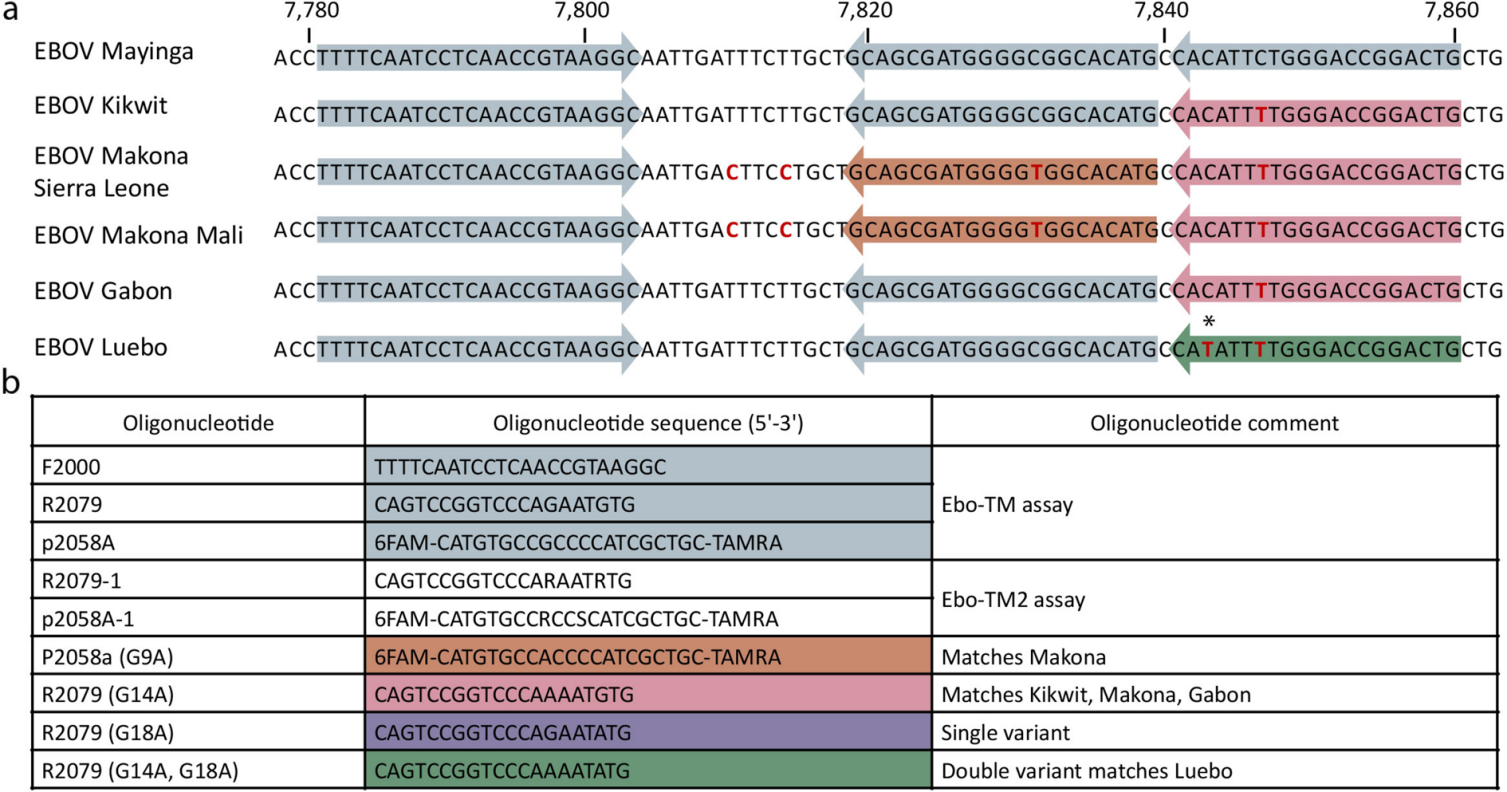
Sequence mismatches identified among EBOV variants. (**A**) Multiple EBOV variants including Mayinga (GenBank #NC_002549), Kikwit (#KR867676), Makona Sierra Leone (#KM233075), Makona Mali (#KP260799), Gabon (#KC242792), and Luebo (#KC242785) were aligned. Shown is the assay target region with the forward and reverse primers indicated by the outer primers and the probe by the inner arrows. (**B**) Primer and probe sequences of primers used throughout the manuscript. Note the color schemes for the primers and probe in (**A**), (**B**) correspond to primer matches with the respective EBOV variant. For example, the green R2079 (G14A, G18A) matches with EBOV Luebo and is shown in (**A**) as the green reverse primer. The R2079 (G18A) primer contains a single, isolate variant indicated by a * in the reverse primer of EBOV Luebo in (**A**).

All of the available EBOV sequences in GenBank at the start of this study (n = 1996) were aligned (settings included: gap open cost = 10.0, gap extension cost = 1.0, end gap cost free, and alignment very accurate) and analyzed (Find Binding Sites and Create Fragments tool for primers and probes) using CLC Genomics Workbench. No forward Ebo-TM primers required modification. Reverse primers and probe were modified as detailed in Fig. 1. Probe G9A was modified to be an exact match to EBOV Makona. Reverse primer G14A was modified to be an exact match to EBOV Kikwit and Makona. Reverse primer G14A, G18A was modified to be an exact match to EBOV Luebo, and reverse primer G18A incorporated the mismatch at the 3’ end of the reverse primer for EBOV Luebo. In order to assess the impact the nucleotide mismatches had on assay performance, the Ebo-TM assay was run as previously described^15^ with different combinations of the primer and probe.

### Real-time RT-PCR

Briefly, 5 µl of extracted nucleic acid from EBOV Kikwit, Mayinga, Makona, and Luebo were assayed using the original Ebo-TM as developed or modified by having (1) the probe switched with probe G9A, (2) Ebo-TM with reverse primer G14A, and (3) Ebo-TM assay with reverse primer G14A, G18A. Assays were run on the Roche LightCycler 480 (Roche Applied Science, Indianapolis, IN) using the SuperScript One-Step RT-PCR Kit (Thermo Fisher Scientific). Cycling conditions were: 50 °C for 15 min; 95 °C for 5 min; 45 cycles (95 °C × 5 s, 60 °C × 20 s); and 40 °C × 30 s. Fluorescence readings were taken following each of the 45 cycles, and a sample was considered positive if the quantification cycle (Cq) was less than 40 cycles. The difference between the Ebo-TM assay and the modified assay (ΔCq) was determined.

Based on the sequence analysis, a new reverse primer (R2079-1, 5′-CAGTCCGGTCCCARAATRTG) and probe (p2058A-1, 5′- 6FAM-CATGTGCCRCCSCATCGCTGC-TAMRA) were designed. This probe had an additional degenerate nucleotide (the R) that was identified in several newly sequenced EBOV isolates submitted to GenBank after the analysis above was completed. The newly optimized assay was run as described above with the original forward primer. Preliminary LODs using both the Ebo-TM assay and the new EBO-TM2 assay were determined using EBOV Makona, Kikwit, Mayinga, and Luebo. Virus was serially diluted 1:10 in nuclease-free water, and the preliminary LOD was the lowest concentration in which all three test replicates were positive. The preliminary LOD was then confirmed by running 60 replicates for each virus at the preliminary LOD. If at least 58/60 replicates were not positive, 60 replicates were repeated at a higher virus concentration until at least 58/60 replicates tested positive.

Exclusivity testing was conducted using the following organisms at 100 pfu/reaction: Sudan virus [Boniface (UCC# R4142S) and Gulu (UCC# R4389T)], Reston virus H28 (UCC# R4387T), Tai Forest virus (UCC# R4371T, GenBank ID MH121167.1), Bundibugyo virus Uganda (UCC# R4386T), Marburg virus [Musoke (UCC# R4383T), Ci67(UCC# R4250T), Ravn (UCC# R4374T), and Angola (UCC# R4185T)], Lassa virus Josiah [(UCC# R4086T), Weller (UCC# R3983a-T), Macenta (UCC# R4341T), and Pinneo (UCC# R4304T)], Mobala virus (UCC# R4043T), Mopeia virus Mozambique (UCC# R3993a-T), dengue virus [serotype 1 (WestPac, UCC# R4423), 2(S16803, UCC# R4424), 3 (CH53489, UCC# R4425), and 4 (341750, UCC# R4426)], yellow fever virus (UCC# R4318T), Rift Valley fever virus (UCC# RV004a-T), West Nile virus [EG101 (UCC# R4310T) and NY99 (UCC# R4272T)], Chikungunya virus 008 (UCC# R4215T) and B (UCC# R4261T), and Crimean-Congo hemorrhagic fever virus IbAr10200 (UCC# R4401T).

### Statistics

GraphPad Prism 5 was used to assess the significance of the impact nucleotide mismatches had on detection. Significance was determined using a t-test (Holm-Sidak method), and a comparison was considered significant if the difference was less than 0.05.

## Results

### EBOV assay analysis

Our group previously developed several assays (Ebo-MGB and the Ebo-TM) targeting the EBOV nucleoprotein (NP) and glycoprotein GP genes, respectively^15^. New EBOV genomic information since the design and characterization of these assays during the 2013–2016 EBOV outbreak in West Africa raised concerns about the impact of genetic diversity on assay performance. An analysis of all EBOV sequences available at the time identified mismatches within the assay target region (Fig. 1) for the Ebo-TM assay. This analysis identified a single mismatch in the assay probe region (G9A in reference to EBOV Mayinga) and two in the reverse primer region (G14A and G18A). No mismatches were identified in the forward primer.

### Sequence variance impact on assay performance

**Figure 2:**
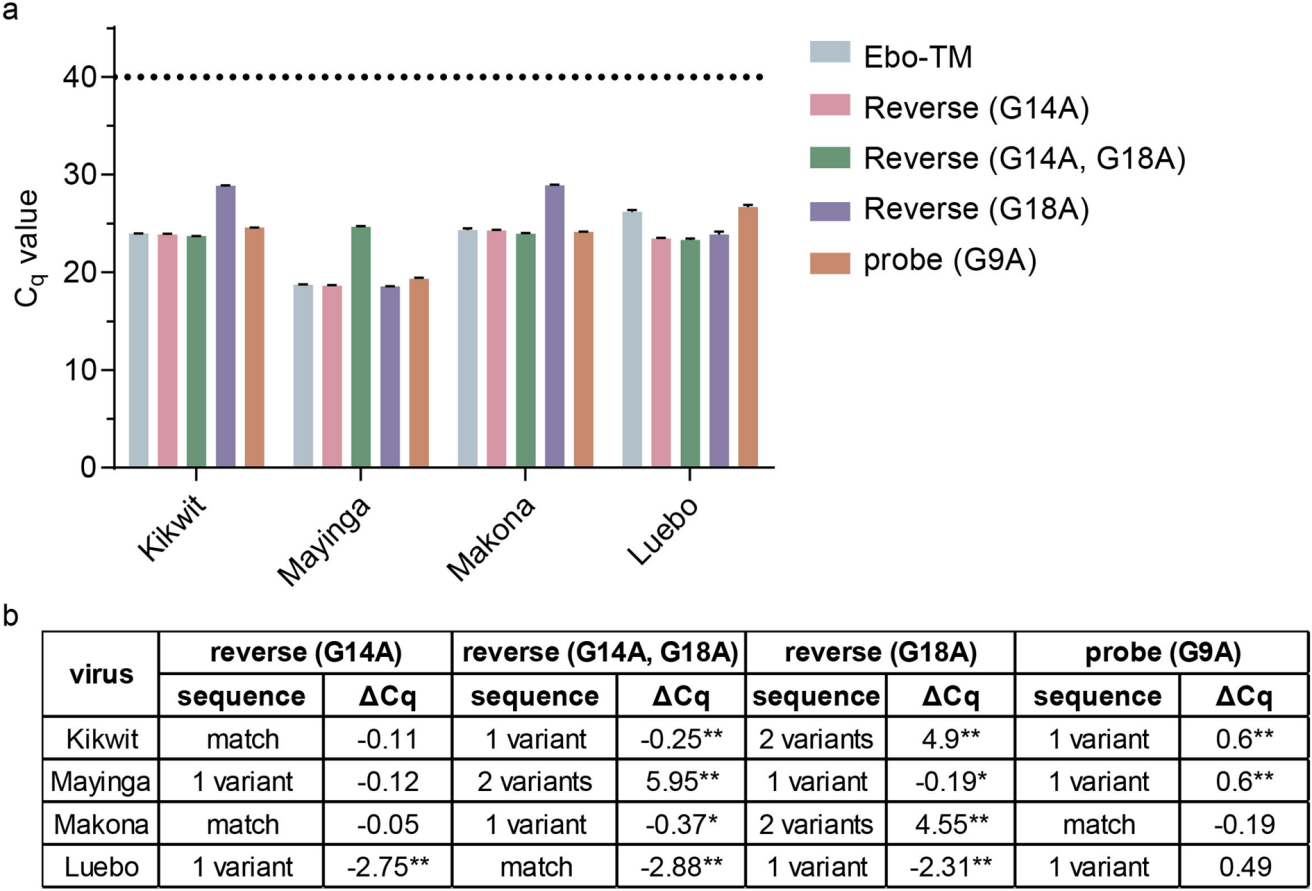
Primer and probe mismatch analysis identified impact of sequence mismatches on target detection. (**A**) EBOV variants Kikwit, Mayinga, Makona, and Luebo were tested by RT-qPCR using different primer and probe combinations identified in Fig. 1. Reported are the Cq values for each assay combination tested with nucleic acid from each EBOV variant. Samples were tested in triplicate and error bars indicate standard deviation. The bar colors correspond to the primers and probe shown in Fig. 1A. (**B**) Changes in real-time PCR signal (ΔCq) was calculated with regard to the Ebo-TM assay. Differences in the primer and probe with regard to the template are shown as a match, a single variant, or a double variant. Significance was determined using a t-test (Holm-Sidak method), and a comparison was considered significant if the difference was less than 0.05 (*p < 0.05, **p < 0.01).

A series of primers and probes (see Fig. 1) corresponding to the different EBOV variants identified were used to determine what effects the mismatches within the Ebo-TM target had on assay performance. Five different assays were tested: 1) the unmodified Ebo-TM (matches Mayinga); 2) Ebo-TM reverse (G14A) that matches Gabon, Kikwit, and Makona; 3) Ebo-TM reverse (G14A, G18A) that matches Luebo; 4) Ebo-TM reverse (G18A) that isolated the change seen in the EBOV Luebo variant from G14A; and 5) Ebo-TM probe (G9A) that matches Makona. Each of the five assays were tested with EBOV Kikwit, Mayinga, Makona, and Luebo. Figure 2A (and Supplementary Fig. 1) show the RT-qPCR Cq values for each assay with each the different EBOV variants with Fig. 2B indicating the impact the different primers and probe have with reference to the Ebo-TM assay.

A single mismatch between primer and template had a minor impact (< 0.5 Cq change) on assay sensitivity while two mismatches resulted in a significant impact (> 4.5 Cq change). For example, the reverse primer in assay Ebo-TM reverse (G18A) had two mismatches for Kikwit and Makona, resulting in a significant 4.9 and 4.55 Cq decrease in sensitivity, respectively. A single mismatch in the probe resulted in an approximate 0.5 Cq change. The assay using Ebo-TM probe (G9A) resulted in a single mismatch for EBOV Kikwit and a match for Makona had a significant 0.6 Cq increase (i.e. reduced assay sensitivity) and a nonsignificant − 0.19 Cq change (i.e. increased assay sensitivity), respectively.

### Assay performance

**Figure 3:**
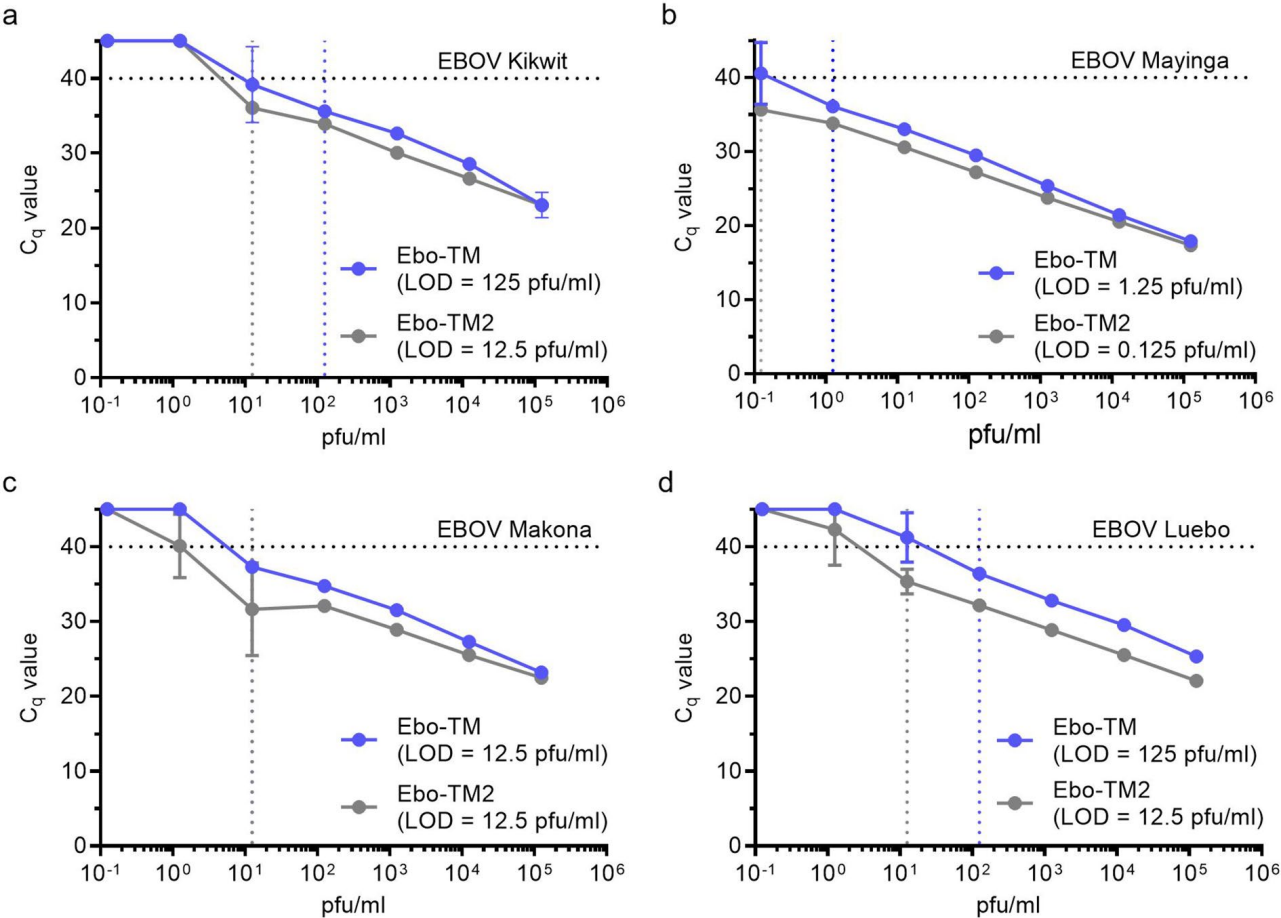
Preliminary LOD identify improved sensitivity for the sequence optimized EBOV assay. Preliminary LODs were determined using serial dilutions of EBOV variants Kikwit (**A**), Mayinga (**B**), Makona (**C**), and Luebo (**D**). Extracted nucleic acid was serially diluted 1:10 in nuclease-free water and assayed by the Ebo-TM assay and the sequence optimized EBO-TM2 assay. Samples were run in triplicate, and the preliminary LOD was lowest concentration where all three replicates were positive. Error bars indicate the standard deviation.

We re-designed the Ebo-TM assay to account for all currently established EBOV variance within the assay target region by incorporating degenerate nucleotides to mitigate the mismatches impact on assay performance (Fig. 1). Assay performance using four different EBOV variants (Kikwit, Mayinga, Makona, and Luebo) showed improved sensitivity, reflected in lower Cq values, for each virus dilution when compared to the original assay (Fig. 3). The Ebo-TM-2 assay resulted in greatest preliminary LOD improvement with EBOV Luebo detection across all replicates at 12.5 PFU/ml compared to 125 PFU/ml for the original assay. Confirmation of this preliminary LOD, in which 60 replicates are repeated at increasing virus concentrations until at least 58 replicates test positive (or 95% success with 90% confidence based on binomial statistics), maintained the improved performance of the new EBO-TM2 assay.

Inclusivity testing showed the new EBO-TM2 assay detected all known EBOV variants (Table 1). An in silico analysis found this new assay showed 100% identity to 99.7% of all EBOV sequences in GenBank (n = 2313). Allowing for a less stringent in silico analysis (allowing for one nucleotide mismatch in the primers and probe while requiring an exact match at the last five nucleotides of the primer’s 3’ end and the first five nucleotides of the probe’s 5’ end), the new assay would detect the remaining 0.3% of the EBOV sequences. This analysis identified a single isolate of EBOV Beni from the recent outbreak in the Democratic Republic of the Congo having a mismatch in the forward primer region. We evaluated assay performance using synthetic RNA representing this variant to ensure accurate amplification. Comparing the Ebo-TM and EBO-TM2 assays with this synthetic RNA found comparable detection (Table 1). Exclusivity testing ensured the developed assay did not yield false positive, non-specific results with near neighbor and/or co-circulating pathogens within similar geographies. This testing showed assay specificity through negative results for a variety of different pathogens including Lassa virus and the related filoviruses Sudan virus, Bundibugyo virus, and Marburg virus (data not shown).

**Table 1:** Confirmation of assay LODs.

Template	Ebo-TM				EBO-TM2			
	LOD (pfu/ml)	Positive	Ave Cq	STDEV	LOD (pfu/ml)	Positive	Ave Cq	Stdev
Kikwit	100	60/60	33.53	0.29	20	59/60	35.64	1.66
Mayinga	1	60/60	35.16	0.44	1	60/60	33.85	0.29
Makona	10	58/60	36.69	0.96	10	59/60	35.01	0.85
Luebo	100	60/60	36.32	0.65	20	60/60	33.76	0.5
Beni	500^a^	60/60	30.99	0.15	50^a^	60/60	33.70	0.38

^a^The LOD for EBOV Beni is in synthetic RNA copies per real-time PCR reaction.

### Clinical sample testing

Analytical testing of the new EBO-TM2 assay indicated improved performance across all EBOV variants, including the EBOV Makona variant testing (Fig. 3; Table 1).  In further testing, we assessed whether this improvement would impact EBOV detection in clinical samples to determine the clinical utility of the assay. Testing with the Ebo-TM and the EBO-TM2 assays across a panel of 21 positive and negative human serum samples collected during the EBOV outbreak in Sierra Leone showed improved sensitivity in clinical samples. All 12 of the samples previously positive by the Ebo-TM assay were also positive using EBO-TM2; however, all but one of the samples had a lower Cq value with the EBO-TM2 (Table 2). Interestingly, three samples had replicates that tested positive using EBO-TM2 but not with EBO-TM (Table 2). One sample had all three replicates test positive while the other two samples had positive and negative replicates indicating an indeterminate test result requiring additional testing for confirmation.

**Table 2:** Clinical sample testing comparing the Ebo-TM and optimized EBO-TM2 assays.

Sample	Ebo-TM		EBO-TM2		ΔCq
	Positive	ave Cq	Positive	ave Cq	
Sample 1	0/3		0/3		
Sample 2	0/3		0/3		
Sample 3	3/3	30.03	3/3	30.8	0.77
Sample 4	3/3	21.11	3/3	20.15	− 0.96
Sample 5	0/3		1/6	38.1	
Sample 6	0/3		0/3		
Sample 7	0/3		3/3	35.84	
Sample 8	3/3	28.3	3/3	27.4	− 0.9
Sample 9	3/3	24.16	3/3	22.99	− 1.17
Sample 10	3/3	21.83	3/3	20.91	− 0.92
Sample 11	3/3	19.49	3/3	18.46	− 1.03
Sample 12	3/3	26.15	3/3	25.31	− 0.84
Sample 13	3/3	27.54	3/3	26.39	− 1.15
Sample 14	3/3	25.18	3/3	24.33	− 0.85
Sample 15	3/3	25.45	3/3	24.39	− 1.06
Sample 16	3/3	27.89	3/3	27.56	− 0.33
Sample 17	0/3		0/3		
Sample 18	0/3		0/3		
Sample 19	0/3		3/6	36.34	
Sample 20	3/3	23.79	3/3	23.06	− 0.73
Sample 21	0/3		0/3		

## Discussion

Well-designed assays are a critical component for infectious disease diagnostics. This is especially true for molecular assays such as real-time PCR where mismatches between the primer/probe and the target organism can have a significant impact on assay performance^14,20^. Designing assays for rare infectious diseases such as EBOV is challenging due to limited availability of genetic information and increasing the risk of false-negative test results due to undiscovered genetic diversity.

Here, we characterized the impact of known primer/probe mismatches on assay performance. Overall, a single mismatch had minor impact while two mismatches had nearly 2-log impact on assay sensitivity. We re-optimized the assay using all known sequence diversity, finding improved sensitivity for all four EBOV variants we tested. The improvement with EBOV Makona was slight but potentially impactful: several Ebo-TM negative human clinical samples tested positive with the sequence-optimized assay. Theoretically, a more sensitive assay would better detect early infections, leading to more rapid isolation and treatment, and more effective identification of viral loads for patient release from isolation wards.

Also of interest is the potential impact viral quasispecies might have in the modest sensitivity improvement observed when a mistmatch was introduced. For example, the original Ebo-TM assay was an exact match to EBOV Mayinga; however, introducing a single variant in the primer decreased the Cq slightly. We hypothesize a small viral population within the EBOV Mayinga stock could have the variants captured within the G14A and G18A primers, resulting in a slight improvement in amplification efficiency and a lower Cq value. This improved sensitivity was also observed with EBOV Mayinga and the EBO-TM2 assay when testing serial dilutions of the virus with the old and new assays.

Warranting further study is the locations of the nucleotide variants and assay performance. While having an exact primer match for EBOV Luebo resulted in a significant increase in sensitivity, the decrease was more noticeable when the variant was closer to the 3’ end of the primer (primer G14A) compared to the variant further away (primer G18A). While the mismatch closer to the 3’ end of the primer (G14A) should theoretically have a greater impact on efficient amplification, it is possible the mismatch in the middle of the four T bases within EBOV Leubo (G18A) has a greater destabilizing effect.

Taken together, these data highlight the importance of appropriately designing assays using as much available sequence information as well as routinely checking assay design for rare pathogens. A deeper sequence availability would help identify more conserved regions for improved assay design. The intended use of the assay is another important consideration in selecting the most appropriate assay. For example, incorporating degenerate nucleotides in the primers/probe would be appropriate for diagnostic assays where improved inclusivity is more important that the most sensitive assay. However, using an assay that is an exact match to a characterized virus challenge stock (and theoretically more sensitive) would be more advantageous for animal model development and vaccine/therapeutics testing.

### Supplementary Information

Supplementary Information.

## References

[1] Dowell SF (1999). Transmission of Ebola hemorrhagic fever: A study of risk factors in family members, Kikwit, Democratic Republic of the Congo, 1995: Commission de Lutte contre les Epidemies a Kikwit. J. Infect. Dis..

[2] Centers for Disease, C. & Prevention. Outbreak of Ebola viral hemorrhagic fever--Zaire, 1995. *MMWR Morb. Mortal. Wkly. Rep.* **44**, 381–382 (1995). 7739512

[3] Ebola haemorrhagic fever in Zaire, 1976. *Bull. World Health Organ.* **56**, 271–293 (1978). PMC2395567 307456

[4] Baize S (2014). Emergence of Zaire Ebola virus disease in Guinea. N. Engl. J. Med..

[5] Leroy EM (2009). Human Ebola outbreak resulting from direct exposure to fruit bats in Luebo, Democratic Republic of Congo, 2007. Vector Borne Zoonotic Dis..

[6] Outbreak news. Ebola virus haemorrhagic fever, Democratic Republic of the Congo-update. *Wkly. Epidemiol. Rec.* **82**, 345–346 (2007). 17918654

[7] Organization, W. H. (2015).

[8] Greiner AL (2015). Addressing contact tracing challenges-critical to halting Ebola virus disease transmission. Int. J. Infect. Dis..

[9] Rivers CM, Lofgren ET, Marathe M, Eubank S, Lewis BL (2014). Modeling the impact of interventions on an epidemic of ebola in sierra leone and liberia. PLoS Curr..

[10] Gsell PS (2017). Ring vaccination with rVSV-ZEBOV under expanded access in response to an outbreak of Ebola virus disease in Guinea, 2016: An operational and vaccine safety report. Lancet Infect. Dis..

[11] Henao-Restrepo AM (2017). Efficacy and effectiveness of an rVSV-vectored vaccine in preventing Ebola virus disease: Final results from the Guinea ring vaccination, open-label, cluster-randomised trial (Ebola Ca Suffit!). Lancet.

[12] Green A (2018). Ebola outbreak in the DR Congo: Lessons learned. The Lancet.

[13] Nkengasong JN, Onyebujoh P (2018). Response to the Ebola virus disease outbreak in the Democratic Republic of the Congo. Lancet.

[14] Stadhouders R (2010). The effect of primer-template mismatches on the detection and quantification of nucleic acids using the 5' nuclease assay. J. Mol. Diagn..

[15] Trombley AR (2010). Comprehensive panel of real-time TaqMan polymerase chain reaction assays for detection and absolute quantification of filoviruses, arenaviruses, and New World hantaviruses. Am. J. Trop Med. Hyg..

[16] Towner JS, Sealy TK, Ksiazek TG, Nichol ST (2007). High-throughput molecular detection of hemorrhagic fever virus threats with applications for outbreak settings. J. Infect. Dis..

[17] Panning M (2007). Diagnostic reverse-transcription polymerase chain reaction kit for filoviruses based on the strain collections of all European biosafety level 4 laboratories. J. Infect. Dis..

[18] Sanchez A (1999). Detection and molecular characterization of Ebola viruses causing disease in human and nonhuman primates. J. Infect. Dis..

[19] Gire SK (2014). Genomic surveillance elucidates Ebola virus origin and transmission during the 2014 outbreak. Science.

[20] Koehler JW (2018). Sequence optimized real-time reverse transcription polymerase chain reaction assay for detection of Crimean-Congo Hemorrhagic fever virus. Am. J. Trop. Med. Hyg..

