# Supplementary material for: Sequence optimized diagnostic assay for Ebola virus detection
Source: Sci Rep. 2023 Nov 1;13:18840. doi: 10.1038/s41598-023-29390-6 (PMC10620139; doi:10.1038/s41598-023-29390-6)
Supplement: Supplementary file 1 — Supplementary Information. [file 41598_2023_29390_MOESM1_ESM.docx]

**Supplementary Table 1. Primer and probe Cq values from Figure 2.** The individual Cq values from Figure 2 are shown for the respective assay and Ebola virus variants. The color coding indicates the specific primer or probe used and follows the color scheme in Figure 1 and Figure 2.

| **Assay** | **Kikwit** | | | **Mayinga** | | | **Makona** | | | **Luebo** | | |
| --- | --- | --- | --- | --- | --- | --- | --- | --- | --- | --- | --- | --- |
| Ebo-TM | 23.96 | 23.96 | 23.97 | 18.66 | 18.76 | 18.76 | 24.18 | 24.27 | 24.53 | 26.26 | 26.34 | 25.97 |
| Reverse (G14A) | 23.94 | 23.82 | 23.81 | 18.62 | 18.52 | 18.67 | 24.25 | 24.24 | 24.35 | 23.35 | 23.51 | 23.47 |
| Reverse (G14A, G18A) | 23.7 | 23.72 | 23.71 | 24.6 | 24.74 | 24.68 | 24.01 | 23.98 | 23.88 | 23.18 | 23.49 | 23.26 |
| Reverse (G18A) | 28.9 | 28.84 | 28.84 | 18.5 | 18.59 | 18.52 | 28.8 | 28.86 | 28.98 | 24.17 | 23.86 | 23.6 |
| probe (G9A) | 24.58 | 24.55 | 24.56 | 19.27 | 19.46 | 19.25 | 24.13 | 24.12 | 24.17 | 26.44 | 26.75 | 26.86 |
